# Angiomyolipome rénal agressif avec extension à la veine rénale: à propos d'un cas et une revue de la littérature

**DOI:** 10.11604/pamj.2017.28.190.7746

**Published:** 2017-10-31

**Authors:** Aziz El Majdoub, Abdelhak Khallouk, Moulay Hassan Farih

**Affiliations:** 1 Service d'Urologie, CHU Hassan II, Fès, Maroc

**Keywords:** Rein, angiomyolipome, tumeur, veine rénale, Rein, angiomyolipoma, tumor, renal vein

## Abstract

L'angiomyolipome rénal est une tumeur qui est reconnue bénigne. Elle présente trois composantes différentes: musculaire, vasculaire et graisseuse, dont la distribution est variable. Le diagnostic repose sur la mise en évidence de la composante graisseuse intra-tumorale en TDM ou IRM. Exceptionnellement la tumeur peut avoir un caractère agressive avec extension locorégionale et veineuse (la veine rénale et la veine cave inferieure). Nous présentons une observation d'une patiente âgée de 37 ans présentant un Angiomyolipome rénal volumineux avec extension à la veine rénale.

## Abstract (en)

Renal angiomyolipoma is a benign tumor. It is characterized of three different components: muscular, vascular and greasy. Their distribution is variable. Diagnosis is based on the identification of the intratumoral greasy component on CT scan and MRI. In exceptional circumstances, this tumor may be aggressive with locoregional and venous extension (renal vein and inferior vena cava). We here report the case of a 37-year old patient with voluminous renal angiomyolipoma extending to the renal vein.

## Introduction

L'angiomyolipome rénal (AML) est une tumeur reconnu bénigne rare (1-3%) à triple composante graisseuse, musculaire lisse et vasculaire en proportions variables. Classiquement ce type de tumeur s'inscrit dans le cadre de la Sclérose Tubéreuse de Bourneville (STB), L'atteinte rénale est alors multiple et bilatérale. Mais, l'angiomyolipome rénal peut être sporadique, isolé et unilatéral avec une forte prédominance féminine. Le diagnostic repose sur la mise en évidence de la composante graisseuse intra-tumorale en TDM ou IRM. Malgré le caractère généralement reconnu bénin de cette tumeur, elle peut être agressive avec extension locorégionale et veineuse. Nous rapportons un cas d'angiomyolipome avec extension à la veine rénale.

## Patient et observation

**Figure 1:**
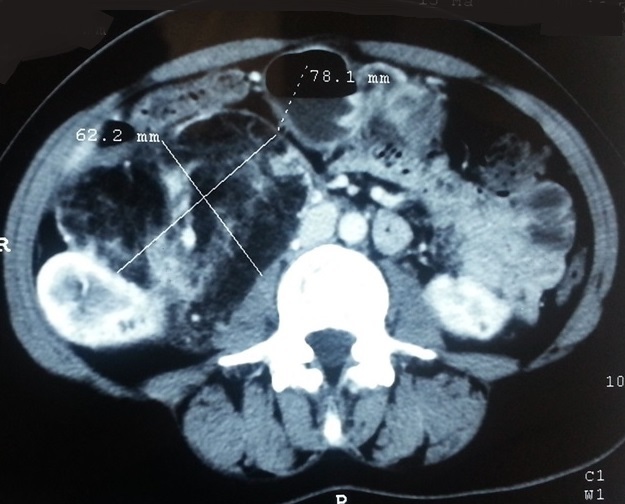
TDM abdominal: coupe axiale mettant en évidence un énorme angiomyolipome du rein droit

Il s'agit d'une patiente, âgée de 43 ans, sans sclérose tubéreuse de Bourneville. L'histoire de la maladie remontait à 6 mois avant la consultation, par l'installation d'une douleur lombaire intense sans irradiation particulière, isolée ne s'associant à aucun autre signe urinaire. L'examen physique était sans anomalies. Une échographie rénale avait objectivé une masse tissulaire solide du rein droit. Une tomodensitométrie abdominale pratiquée pour une meilleure caractérisation de la masse. Elle avait permis la détection d'un énorme syndrome tumoral du rein droit, mesurant 62,2/78,1 mm de grand diamètre. La tumeur présentait un aspect de densité mixte graisseuse et tissulaire prenant fortement le produit de contraste, entrainant la rupture du cortex rénal (Figure 1). Cet aspect scanographique avait fait évoquer Un processus tumoral angiomyolipomateux ou un liposarcome rétropéritonéal. L'indication d'une néphrectomie totale avait été posée vu que l'attitude conservatrice n'était pas possible devant la grande taille de l'AML (80mm) exposant au risque hémorragique et à l'incertitude de la nature bénigne de la tumeur. Les suites post opératoires étaient simples avec un séjour post opératoire de 3 jours. L'étude anatomopathologique était en faveur de l'AML du rein droit qui mesurait 17 cm, par la mise en évidence d'une prolifération tumorale bénigne faite de trois composantes: musculaire lisse fusiforme à noyau globuleux et vésiculeux, vasculaire à paroi épaisse et adipocytaire prédominante. La tumeur infiltrait la paroi de la veine rénale droite avec des marges chirurgicales négatives.

## Discussion

L'angiomyolipome (AML) est une tumeur bénigne (hamartome) qui représente 1 à 3% des tumeurs solides du rein. Cette lésion est composée, dans des proportions variables, d'un contingent graisseux souvent le plus abondant, d'un contingent de cellules musculaires lisses et d'un contingent d'origine vasculaire [1-3] .Classiquement, ce type de tumeur s'inscrit dans le cadre de la Sclérose Tubéreuse de Bourneville (STB). Le caractère invasif de certains angiomyolipomes a été rapportés dans la littérature, il est exceptionnel en dehors d'une forme rare d'angiomyolipome: l'angiomyolipome épithéloide.il s'agit de l'envahissement veineux et de l'atteinte ganglionnaire régionale. L'extension à la veine rénale et à la veine cave inferieure est rare, avec quelques cas cliniques isolés rapportés dans la littérature [4,5]. L'extension est strictement endoveineuse, en continuité avec la lésion rénale sans envahissement des parois vasculaires .l'uroscanner avec mesure de la densité du thrombus et l'IRM en pondération T1 sans et avec suppression du signal de la graisse permettent de faire le diagnostic .en échographie, le thrombus est hyperéchogène , homogène et bien limité [6]. Le principal diagnostic différentiel, certes, est le carcinome du rein à composante graisseuse .les ilots graisseux sont dans ce type de tumeur liés à une métaplasie osseuse associant moelle osseuse et tissu osseux calcifié. En général l'angiomyolipome rénal est considérée comme une tumeur bénigne et est associé à un pronostic favorable. Cependant, un cas rare d'AML rénale présentant une transformation liposarcomateuse a déjà été rapportée [7]. Yiu et al [8] ont résumé seize études et identifié qu'il y a aucune association entre le comportement agressif et la taille de la tumeur. Eble [9] a identifié 20 cas d'AML avec envahissement veineux ou ganglionnaire régional. il a démontré que l'étude anatomopathologique des tumeurs, des ganglions et des thrombus n'a pas objectivé de caractères de malignité et aucun patient n'a présenté une récidive ou progression au cours du suivi. Après un examen de 35 cas dans la littérature, Tan et al [10] a émis l'hypothèse que lorsqu'un AML présente un comportement agressif, la transformation maligne a eu lieu. Le critère qui confirme la caractère malin d'un angiomyolipome rénal est la présence de métastases à distance. Pour la prise en charge thérapeutique, les tumeurs moins de 4 cm de diamètre nécessite une surveillance échographique annuelle. Le but du traitement est de traiter ou d'éviter les complications (hémorragie, douleur) tout en préservant le capital néphrotique. L'embolisation artérielle sélective répond à cet objectif suite au développement du matériel utilisé [11]. La chirurgie est indiqué en cas d'échec de l'embolisation, angiomyolipome avec thrombus de la veine rénale ou de la veine cave inférieure, ou lorsque il existe un doute sur la nature histologique de la tumeur [12]. La tumorectomie est à discuter en fonction des rapports de la tumeur avec le hile rénal et la multiplicité des lésions, alors que la néphrectomie partielle ou totale doit être réservée aux angiomyolipomes compliqués de syndrome hémorragique non contrôlable par l'embolisation ou en cas de suspicion de tumeur maligne .

## Conclusion

L'Angiomyolipome rénal est une tumeur connue généralement bénigne rare, qui présente trois composantes différentes: musculaire, vasculaire et graisseuse. Mais il peut dans certains cas avoir un comportement agressif avec extension veineuse et ganglionnaire. Mais ce caractère n'oriente pas obligatoirement vers la malignité. Ce type de cas nécessite une néphrectomie totale avec thrombectomie pour éviter les complications emboliques à distance.

## Conflits d’intérêts

Les auteurs ne déclarent aucun conflit d'intérêts.
